# Real-Life Outcome in Multiple Sclerosis in the Czech Republic

**DOI:** 10.1155/2019/7290285

**Published:** 2019-02-18

**Authors:** Gisela Kobelt, Linus Jönsson, Miluse Pavelcova, Eva Kubala Havrdová

**Affiliations:** ^1^European Health Economics AB, Stockholm, Sweden; ^2^Department of Neurobiology, Care Science and Society, Karolinska Institutet, Stockholm, Sweden; ^3^Department of Neurology and Center for Clinical Neuroscience, First Medical Faculty, Charles University, Prague, Czech Republic

## Abstract

**Background:**

Cohort studies and registries provide opportunities to estimate long-term outcome in multiple sclerosis.

**Objectives:**

To describe changes in disability (EDSS), relapse activity, and health care consumption over the period 2008-2015 by combining two Czech cost-of-illness studies with disease data from the MS Center in Prague.

**Methods:**

The combined dataset included 426 patients with a mean observation time of 8.3 years. A Cox proportional hazards model with time-varying covariates for treatment, disease course, and EDSS was applied to estimate the effect of treatment on the risk of progression to EDSS 4 and the risk of relapses. The use of health care resources (hospitalization, consultation, and tests) was compared between the two cross-sectional studies.

**Results:**

Total health care costs appeared stable between 2008 and 2015, despite more intense use of disease-modifying treatments in 2015 (52% of patients versus 31% in 2008). 39% of patients starting treatment at EDSS 0-3 in 2008 progressed to EDSS 4 or higher by 2015, while 65% of patients starting at EDSS 0-2 remained stable. The number of relapses was associated with a higher risk of progression. In a marginal structural Cox model of the relapse risk, treatment with natalizumab or fingolimod was associated with a lower risk of relapse (hazard ratio 0.68, p<0.01). Treatment with natalizumab or fingolimod was associated with a lower risk of progression to EDSS 4.

**Conclusion:**

Our results link relapses to progression and indicate that the newer treatments have a better effectiveness, despite difficulties caused by small a sample size, administrative rules guiding treatment, and absence of a random comparator group.

## 1. Introduction

In the past, long-term outcome in multiple sclerosis (MS) has most often been modelled using clinical and natural history data combined with economic surveys, but increasingly registries and cohort studies provide an opportunity to analyse real-life data. Economic studies in MS have a long tradition; the first cost-of-illness paper was published in 1986 and many large-scale surveys have been performed since then [1–3]. Most of these studies were based on self-reported data to ensure capturing both health care and personal costs as well as disease effects and subjective symptoms and health related quality of life.

In 2007, a group of seven MS clinics in the Czech Republic initiated a cost-of-illness study in 909 consecutive patients who visited one of the centers during that year and were willing to participate in the survey (COMS) [4]. Disease data were collected by clinicians during the inclusion visit and patients independently completed a resource use questionnaire similar to the one used in a survey in 10 European countries in 2005 [5]. In 2015, the Czech Republic was included in the update of the 2005 cost-of-illness study (MSCOI) [3], and the Czech analysis including 747 patients was subsequently published separately [6]. Both surveys ensured that patients at all levels of disability were represented. A comparison of annual direct health care costs per patient (expressed in 2015 CZK) in the two studies showed that these were stable at slightly less than 200,000 CZK (€ 7,750), despite an increased use of disease-modifying treatments (DMT) in 2015. Thus, it is interesting to investigate differences in patients who had participated in both studies.

During data collection in 2015, an effort was therefore made to identify respondents in COMS still followed regularly at the MS Center of Charles University (Prague), with the intention to describe the development of disability and resource consumption for this group during the follow-up period. However, the information provided by two data points is limited. In particular the analysis of outcomes such as relapse activity or effectiveness of treatments requires longitudinal data. Consequently, the information was complemented with data from the clinic database of the MS Center, with the objective to investigate disability development, relapse activity, and the effectiveness of treatment, as well as health care consumption.

## 2. Methods

### 2.1. Patients

The invitation to participate in the burden of illness survey in 2015 was distributed by two Czech patient associations to all of their members. Responses to the questionnaire could be given anonymously either online or by returning a paper copy to the patient organization [3, 6]. Simultaneously the MS Center in Prague contacted all patients still followed at the Center who had earlier participated in COMS by telephone. Patients who intended to participate in the new survey were asked to indicate their COMS identification number in their answer to allow comparison within the two surveys. Of the 482 patients contacted, 426 responded to the survey in 2015. The identities of the patients were solely known to the MS Center. We extracted all available information on disability, relapses, and treatments for these patients from the database of the MS Center in October 2017. Our sample represents thus a group of patients in routine clinical management in Prague, some of them since 1996, who had also been recruited into COMS study in 2008 and answered the 2015 survey. In addition, these patients were also followed in the Czech MS Registry, which ensured standard follow-up and, in particular, annual EDSS assessment.

The dataset for analysis contained longitudinal data on disease course, EDSS scores, relapses, and treatment between 1996 and 2017 originating from the MS Center, as well as EDSS scores, EDSS at treatment start, and health care consumption (hospitalization, consultations, tests, and disease-modifying treatment use) at two data points (2008; 2015) from COMS and MSCOI.

### 2.2. Comparison of the COI Data

Due to differences in some of the nonhealth care resource use questions in the two surveys, the comparison was limited to inpatient care, consultations, investigations, and disease-modifying treatments (DMTs). Costs from COMS were adjusted to CZK 2015 using the consumer price index (CPI).

In addition, considering the importance given today to early treatment, we explored differences in EDSS development depending on when treatment was initiated. The sample on treatment since 2008 was however small (n=132) and results are indicative only.

### 2.3. Treatment Effectiveness

A Cox proportional hazards model with time-varying covariates for treatment, EDSS scores, and disease course (CIS, RRMS, PRMS, SPMS, and PPMS) was used to estimate the effect of treatment on the risk of progression to EDSS 4 and the risk of relapses. Treatments were categorized in three groups: (1) gammaglobulins, azathioprine, and cyclophosphamide, (2) beta-interferons and glatiramer acetate, (3) natalizumab and fingolimod. For each patient follow-up time was segmented according to start and end dates of treatment periods; periods of combination therapy were allocated to the most intense treatment received.

To account for time-varying confounding, observations were weighted according to the probability of receiving the allocated treatment. Stabilized weights were calculated based on treatment probabilities estimated using an ensemble classifier (random forest) [7].

(1) $$\begin{array}{c} {SW}_{it}=\overset{m}{\underset{t=1}{\prod }}\frac{f\left(A_{t}\;|\;{\overset{-}{A}}_{t-1},\overset{-}{V}\right)}{f\left(A_{t}\;|\;{\overset{-}{A}}_{t-1},{\overset{-}{L}}_{t-1}\right)} \end{array}$$

In the nominator, stabilization weights were estimated based on treatment history A_t-1_ and baseline variables V (gender; age of onset). The denominator of the weights was estimated based on prior treatment history A_t-1_ and covariate history L_t-1_

Estimated treatment probabilities close to zero or one were truncated so that 0.01<p<0.99, in order to avoid variance inflation due to very large weights [8]. The only right-censoring of data was administrative; thus no correction for informative censoring was done.

In the Cox model of relapse risk, multiple failures (relapses) per subjects were allowed. Standard errors were estimated with the Sandwich Variance estimator [9].

## 3. Results

### 3.1. Comparison of the Two Surveys

The demographics and resource consumption of the sample at the time of COMS and MSCOI are presented in Table 1. DMT use increased from 31% to 52%, with over half of the patients between EDSS 0 and 6.5 on treatment in 2015. Other health care resources, however, were used less.

For the sample overall, costs of hospitalization, consultations, and tests during the quarter preceding the data collection were 40% lower in 2015 than in 2008. Half of the reduction was for tests, while hospitalization represented 30% and consultations 20% of the difference. The reduction was most pronounced in the mild group, while costs in the severe group increased.

Although the mean EDSS score for this group of 426 patients advanced only slightly over the 8 years (from 3.0 to 3.2), disease progression is illustrated by the transitions to more severe disease states levels: 77% of patients had an EDSS score below 4 in 2008 compared to 58% in 2015. The group who remained on treatment during the entire period had a stable EDSS score. Of patients who started treatment at EDSS 0 or 1, 61% remained at that level after 8 years; of those starting treatment at EDSS 2, 65% remained at EDSS 2 or better. On the other hand, 70% of patients who had started treatment at EDSS 3 progressed to EDSS 4 or higher, and 75% of those starting at EDSS 4 progressed to EDSS 5 or higher.

### 3.2. Effectiveness of Treatment

The 426 patients were followed on average 8.3 years. The mean EDSS score at inclusion in the database was 3.0, and 311 patients (73%) were below EDSS 4.

**Figure 1:**
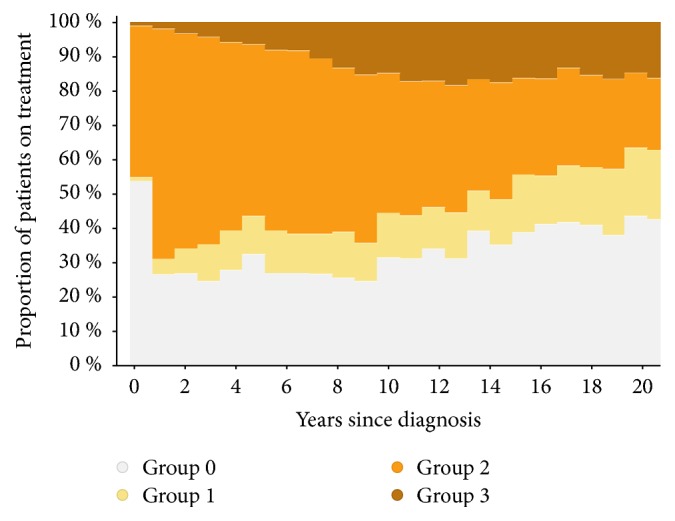
*Proportion of the sample on treatment and type of treatment since disease onset. *The vast majority of patients at onset were treated with group 2 treatments (interferons/glatiramer acetate), with a gradual switch to group 3 (natalizumab/fingolimod). The proportion of patients using group 1 drugs alone is small and remains stable. (Groups 2 and 3 may include patients on combination therapy with group 1, while combination of groups 2 and 3 is very rare.)

**Figure 2:**
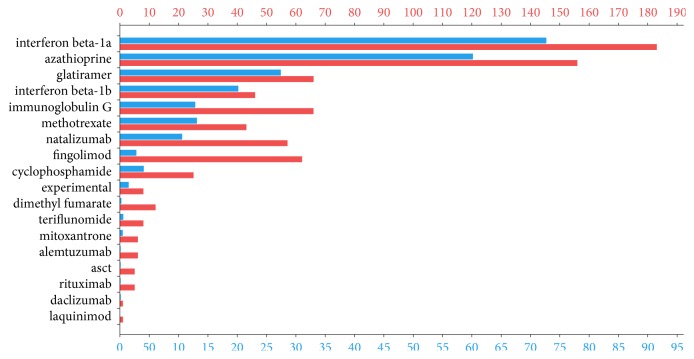
*Total number of patients and total treatment duration in years by single drugs.* Blue bars indicate the total number of patients ever exposed to each drugs during the follow-up; red bars indicate the cumulative treatment time for each drug.

There were in total 732 treatment-years in 162 patients with immunoglobulins, azathioprine, methotrexate, or cyclophosphamide (group 1), 1758 treatment-years in 230 patients with interferons or glatiramer acetate (group 2), and 474 treatment-years in 105 patients with natalizumab or fingolimod (group 3).  Figure 1 shows the proportion of the sample treated with the three DMT categories over time since disease onset, and Figure 2 shows the total number of patients and total treatment exposure by single drugs. The use of the newer generation of drugs appears still limited, as a consequence of the reimbursement requirement of two relapses to switch until 2016 when fingolimod was allowed after 1 relapse.

Without adjusting for confounding, the average annual number of relapses was 0.13 for untreated patients, 0.19 for IgG, 0.40 for interferons and glatiramer acetate, and 0.28 for natalizumab and fingolimod. In a marginal structural Cox model of the relapse risk, treatment with natalizumab or fingolimod was associated with a lower risk of relapse (hazard ratio 0.68, p<0.01). The hazard ratios for treatment groups 1 and 2 were 0.77 (p=0.08) and 0.94 (p=0.55), respectively.

**Figure 3:**
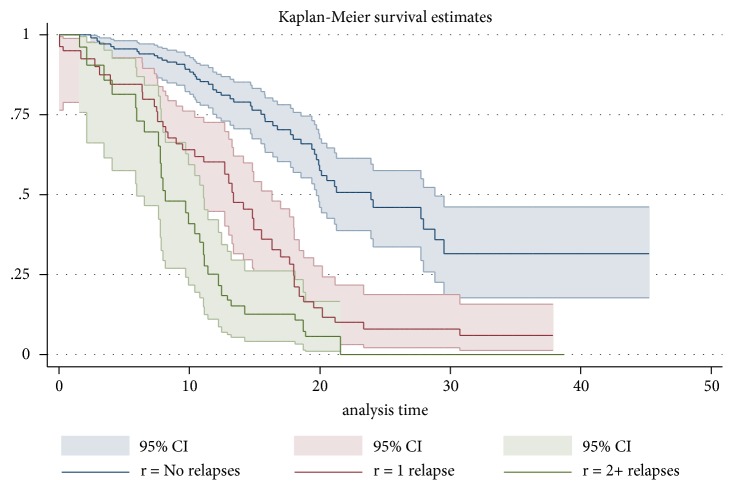
*Progression to EDSS 4 by number of relapses (N=311).* The analysis includes all 311 patients with an EDSS<4 at baseline, regardless of treatment status.

Among the 311 patients with EDSS<4 at baseline, 121 patients (39%) progressed to EDSS 4 during the course of the study. The number of relapses during the previous year was significantly associated with higher risk of disease progression (p<0.001, log-rank test for equality of survival curves) (Figure 3). Natalizumab or fingolimod (group 3) were associated with a lower risk of relapse, in both the IPTW weighted (hazard ratio 0.682) and unweighted (hazard ratio 0.929) analyses, than treatments in group 1 or 2 (reference no treatment) (Table 2).

**Figure 4:**
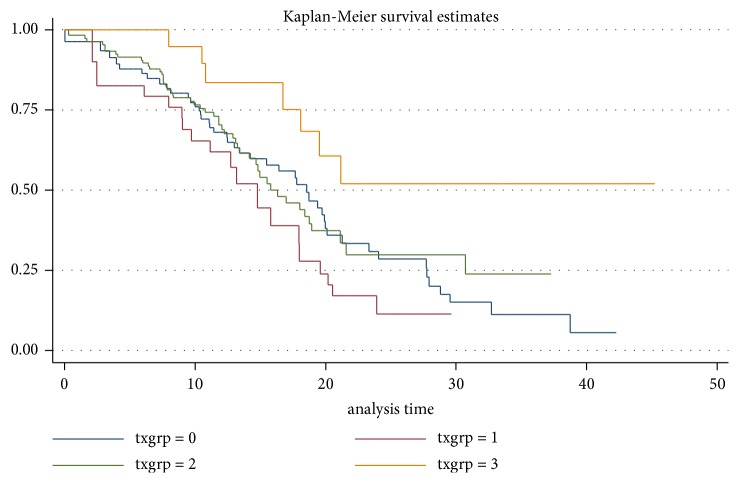
*Progression to EDSS 4 by treatment group (unadjusted).* The unadjusted analysis includes all treatment periods. Groups 0-2 are not significantly different, while group 3 appears to progress slower. However, the sample in group 3 is small (91 patients). txgrp 0 = no treatment; txgrp 1 = gammaglobulins, azathioprine, and cyclophosphamide; txgrp 2 = interferons and glatiramer acetate; txgrp 3 = natalizumab and fingolimod.

Progression to EDSS 4 appeared not different for patients not on treatment or treated with group 1 or 2 (Figure 4). Treatment with natalizumab or fingolimod (group 3) was associated with a lower risk of progression to EDSS 4 (hazard ratio 0.45, p=0.07 in the IPTW weighted analysis, and 0.36 p=0.02 in the unweighted analysis) (Table 3).

## 4. Discussion

Outcome in MS in clinical practice needs to be analysed over the long term due to the generally slow progression of the disease to worse health states. Extrapolating short-term observational or clinical trial data, while being necessary for instance at introduction of new products, will not provide the answer, as patients switch between treatments or their disease management and external conditions change. This analysis combines a real-life database and survey data spanning over 8 years to explore disease development, treatment outcome, and costs.

Despite the limited sample of 426 patients, the results confirm that the number of relapses impacts progression and that the newer DMTs achieve a better outcome as they have a better effect on relapse activity. A number of issues in this analysis require discussion.

Relapses have long been the only well-known and visible risk factor for progression [10–12]. The main treatments thus focus on avoiding or reducing the number of relapses in the short term, thereby reducing progression in the longer term. DMTs introduced in the past 10-15 years have shown to be more effective in the control of relapse activity. During the time period concerned in our study, natalizumab and fingolimod were the only new DMTs that were reimbursed in the Czech Republic, and more recent arrivals could only be used on an individual basis and are not included in our analysis. Also, in view of the limited number of patients we consider natalizumab and fingolimod as one class. A review of natalizumab revealed a better effectiveness compared to the early DMTs [13] and a retrospective analysis of real-world data from the French MS Observatory (OFSEP) found that the proportion of patients with one relapse or more in the first 2 years of treatment was lower with natalizumab than with fingolimod [14]. On the other hand, a recent systematic review of fingolimod in the real world including 34 studies found that the drug improved outcomes compared to interferons and glatiramer acetate, but conflicting trends when comparing fingolimod to natalizumab [15]. This review highlights the difficulty encountered when comparing real-life studies, which was also pointed out in an analysis of real-world long-term benefits of DMTs [16]. The author concludes that collectively these studies are inconclusive but suggest that a long-term benefit may exist. Importantly, he points to the challenges inherent in establishing meaningful therapeutic benefits in a relatively unpredictable, multiphasic, chronic disease where patients are free to start, switch, and stop treatments. This is also one of the major issues in our analysis.

The rules for reimbursement of DMTs in the Czech Republic are very stringent both when instituting the first treatment and particularly when switching to the newer and more expensive treatments. At the time of this study, switching was only allowed after at least two relapses while being on first-line therapy (http://www.sukl.cz/modules/medication/detail.php?code=0168462&tab=prices) (since mid-2016, switching is allowed after the first breakthrough on first-line therapy for fingolimod, http://www.sukl.cz/modules/medication/detail.php?code=0168462&tab=prices). A review of studies of all DMTs approved in Europe until May 2015 recommends that, in the case of breakthrough on first-line therapy, second-line therapy should be instituted. This is the case in many countries and recommended by the new European treatment guidelines [17]. The finding even in this small sample that relapsing patients progress faster indicates in our view that an earlier switch to more effective treatment is preferable in the long term.

Similarly, during the years of follow-up covered by our analysis, DMT treatment could only be instituted in Czech patients with a confirmed diagnosis (http://www.sukl.cz/modules/medication/detail.php?code=0027262&tab=prices); patients with a clinically isolated syndrome (CIS) had no access. Currently, the data arguing for early treatment are rich and convincing [18], and this restriction has been lifted in 2009. While it was not possible to investigate the effect of starting treatment after a single CIS, our exploratory analysis of transitions between EDSS states at the times of the two cost-of-illness surveys, regardless of the type of treatment, points to a better effect of early treatment.

The analysis of treatment effectiveness in this study is complicated by this fact that administrative rules drive therapy with no random allocation to treatment. There are differences in the disease between the patients on the different classes of DMTs, with only patients with the most active disease on second-line drugs, and predominantly patients with inactive and benign disease not on treatment. We have attempted to control for this in our analysis of progression (time to EDSS 4) by weighting observations by the inverse probability of treatment, thus reducing bias due to observed confounders. Despite this, the time to EDSS 4 for untreated patients and patients on first-line treatment is not significantly different, although there is a trend for the interferon group to progress faster. This is typically explained by the selection of more severe patients for treatment.

Overall, disease progression was comparable to that found in other studies. Median time to EDSS 4 was 17.7 (14.8-19.4) years. Comparing to the estimates for patients with relapsing-remitting MS at onset and not on DMTs from the EDMUS cohort from Lyon (France) [10], patients in COMS reach EDSS 4 considerably later (median time 17.7 compared to 11.4 years). One might argue that this constitutes at least partially a treatment effect. The number of patients reaching EDSS 6 in our sample was very small and is indicative only. Time to EDSS 6 was, however, comparable to the estimates from Lyon (23.1 years). Also, estimates in different cohorts span from 11 to 30 years, with the longest times found in a recent study from the Swedish [19], and in the population-based cohorts in British Columbia, Canada, and Olmsted County, United States [20–22]. Earlier studies in Canada and France have shown shorter times to EDSS 6 [12, 23–25]. These studies differ in the size of the samples, the length of follow-up, the age of the population at baseline, and proportions on treatment, and it is difficult to draw any conclusions regarding the sample in this study. However, the rather slow overall progression observed may indicate that enrolment into COMS produced a somewhat biased sample, with a focus on early and on mild patients, to institute treatment as early as possible. The fact that even in 2015 there were still close to half of the patients without treatment would indicate such a bias towards benign disease.

Finally, the comparison of costs in our two surveys is hampered by a major methodological difference. Participants in COMS were identified by MS centers based on a consultation in 2007 and included during a baseline visit with a number of tests in preparation for the prospective follow-up. The 2015 survey was random and anonymous. Patients in COMS thus had nonrandom resource consumption, as can be seen in Table 1 where all patients had consultations. This may explain the lower costs of outpatient care seen in 2015. Cross-sectional surveys do not allow concluding on causality. Nevertheless, it is most likely that the reduction in hospitalization follows the general trend over the past two decades to favor outpatient over inpatient treatment. The substantial decrease in consultations and tests on the other hand can be explained by a methodological difference in the two surveys. COMS recruited patients during a visit; i.e., patients were selected on the dependent variable and 100% of participants had a visit during the past 3 months and almost all also underwent a certain number of tests. In 2015, patients answered by mail or electronically, and consumption represents thus routine management.

## 5. Conclusions

Our study illustrates a number of the difficulties that arise when analysing longitudinal data or comparing costs in surveys in MS. Due to the slow evolution of the disease over many years, progression to more advanced disability states is difficult to observe and samples become very small. This is, however, not different from clinical trials, where progression has to be mostly extrapolated. The major difficulty for analysing treatment effect—in addition to identifying a relevant untreated comparison—resides in the administrative rules imposed treatment. These rules also affect costs. However, although the two surveys in our study used the same questionnaire for resource consumption, the difference in the mode of recruiting patients resulted in a major difference in costs.

## Figures and Tables

**Table 1:** Demographics and resource consumption (*N* = 426).

	2008	2015
Mean age	40	47

Age at first symptoms	28	28

Age at diagnosis	32	32

Disease duration (years)	8	15

Education		
(i) High school/vocational	61%	61%
(ii) University	33%	33%

Employment	57%	53%
(i) full time	53%	0%

DMT treatment	31%	52%
(i) EDSS 0-3.5	27%	60%
(ii) EDSS 4-6.5	13%	51%
(iii) EDSS 7-9	3%	20%

Resource use		
(i) Hospitalization	10%	2%
(ii) Consultations	100%	57%
(iii) Tests	82%	35%

COMS: DMT: disease modifying treatment; EDSS: expanded disability status scale.

**(a):** 

Analysis of Maximum Likelihood Estimates								
with Sandwich Variance Estimate, IPTW weighted								
Parameter		DF	Parameter Estimate	Standard Error	StdErr Ratio	Chi-Square	Pr > ChiSq	Hazard Ratio
Treatment group	1	1	-0.26179	0.15279	1.180	2.9357	0.0866	0.770
Treatment group	2	1	-0.05780	0.09597	1.390	0.3627	0.5470	0.944
Treatment group	3	1	-0.38346	0.14685	1.351	6.8183	0.0090	0.682

**(b):** 

Analysis of Maximum Likelihood Estimates									
with Sandwich Variance Estimate, Unweighted									
Parameter		DF	Parameter Estimate	Standard Error		StdErr Ratio	Chi-Square	Pr > ChiSq	Hazard Ratio
Treatment group	1	1	0.22118	0.13351	1.162	2.7443	0.0976	1.248	
Treatment group	2	1	0.30767	0.09959	1.237	9.5443	0.0020	1.360	
Treatment group	3	1	-0.07314	0.14171	1.237	0.2664	0.6058	0.929	

**(a):** 

Analysis of Maximum Likelihood Estimates, IPTW weighted							
Parameter		DF	Parameter Estimate	Standard Error	Chi-Square	Pr > ChiSq	Hazard Ratio
Treatment group	1	1	-0.32243	0.41160	0.6137	0.4334	0.724
Treatment group	2	1	0.03347	0.28204	0.0141	0.9055	1.034
Treatment group	3	1	-0.81062	0.45828	3.1288	0.0769	0.445

**(b):** 

Analysis of Maximum Likelihood Estimates, unweighted							
Parameter		DF	Parameter Estimate	Standard Error	Chi-Square	Pr > ChiSq	Hazard Ratio
Treatment group	1	1	-0.15071	0.31195	0.2334	0.6290	0.860
Treatment group	2	1	-0.31822	0.24403	1.7004	0.1922	0.727
Treatment group	3	1	-1.03244	0.42806	5.8173	0.0159	0.356

## Data Availability

The clinical data used to support the findings of this study are restricted by the Ethics Board of Charles University in order to protect patient privacy. Data are available from G.Kobelt (gk@healtheconomics.se) for researchers who meet the criteria for access to confidential data.
